# IGF Pathway Components as Potential Biomarkers in Gastric Cancer

**DOI:** 10.3390/ijms262210880

**Published:** 2025-11-10

**Authors:** Betul Ceylaner, Furkan Sahin, Anil Yildiz, Didem Tastekin, Ali Fuat Kaan Gok, Ahmet Tarik Baykal

**Affiliations:** 1 Department of Biochemistry, Faculty of Pharmacy, Acibadem Mehmet Ali Aydinlar University, Istanbul 34752, Türkiye; 2 Department of Biochemistry and Molecular Biology, Institute of Health Sciences, Acibadem Mehmet Ali Aydinlar University, Istanbul 34752, Türkiye; furkan.sahin1@live.acibadem.edu.tr; 3 Acibadem Labmed Clinical Laboratories, Istanbul 34752, Türkiye; 4 Department of Clinical Oncology, Institute of Oncology, Istanbul University, Istanbul 34116, Türkiye; anilyildiz@istanbul.edu.tr (A.Y.); didem.tastekin@istanbul.edu.tr (D.T.); 5 Department of General Surgery, Surgical Sciences, Istanbul Faculty of Medicine, Istanbul University, Istanbul 34116, Türkiye; afkgok@istanbul.edu.tr

**Keywords:** gastric cancer, IGF signaling, biomarker

## Abstract

Gastric cancer is one of the leading causes of cancer-related deaths worldwide, and due to its late-stage diagnosis, the prognosis is poor. Therefore, researching biomarkers that can be used in early diagnosis and prognostic processes is crucial. Insulin-like growth factor (IGF) signaling pathways play critical roles in cellular proliferation, apoptosis, differentiation, and tumor progression. This study investigated the biomarker potential of IGF-1, IGFBP-4, IGFBP-5, and PAPP-A in gastric cancer. Forty gastric cancer patients and forty healthy individuals were included, and protein levels in serum samples were measured using the ELISA method. The findings showed that IGF-1 levels were significantly decreased in the gastric cancer group, while IGFBP-4 levels were significantly increased. IGFBP-5 levels were also lower in gastric cancer patients. In contrast, PAPP-A levels did not differ significantly between the two groups. ROC analyses revealed that IGF-1, IGFBP-4, and IGFBP-5 have good discriminatory properties in the diagnosis of gastric cancer, while PAPP-A offers low diagnostic value. In conclusion, this study suggests that IGF pathway components, particularly IGF-1, IGFBP-4, and IGFBP-5, might be promising biomarker candidates for gastric cancer.

## 1. Introduction

Gastric cancer (GC) is the fifth leading cause of cancer-related deaths worldwide, with approximately one million new cases and more than 660,000 deaths annually [1]. Gastric cancer is histopathologically divided into two main subtypes, known as intestinal and diffuse types. The intestinal type exhibits a higher incidence rate and is predominantly associated with environmental factors and Helicobacter pylori infection. In contrast, the diffuse type of cancer is characterized by a genetic background and demonstrates a lower incidence rate [2,3]. Since the progression of both types is often asymptomatic, diagnosis is usually made at an advanced stage, which negatively affects survival. The incidence of gastric cancer rises with age, whereas only a small proportion (less than 10%) of patients are diagnosed before the age of 45 [4].

The life cycle of a normal human cell is closely regulated by intracellular and extracellular signals that work in harmony to appropriately control cellular proliferation, senescence, and apoptosis [5]. The insulin-like growth factor (IGF) signaling pathway is one of these signals that plays an important role in cellular proliferation, apoptosis, differentiation, cellular survival, tissue growth, and development [6]. The IGF system consists of receptors (IGF-1R and IGF-2R), ligands that bind to them and activate them (IGF-1 and IGF-2), and IGF-binding proteins (IGFBP-1 to IGFBP-6) that bind IGF proteins. Specific IGFBP proteases called pregnancy-associated plasma protein A (PAPP-A) and its sequence homolog PAPP-A2, and Stanniocalcin 1 and 2 (STC) proteins, whose protease activity is known to be restricted to the Pappalysin metzincin metalloproteinase family [7], are also regulatory molecules of this pathway.

The binding of IGF-1 or IGF-2 proteins to IGF-1R results in a series of cellular events that lead to phosphorylation of the receptor, activation of the intracellular signaling cascade, cellular survival, proliferation, and migration, respectively. In tumor cells, IGF-1R activation can cause angiogenesis, invasion, and metastasis [8]. IGF-1R is known to suppress autophagy by activating the Ras/Raf/MEK and PI3K/Akt/mTOR pathways, thereby contributing to proliferation, and increasing cell migration and metastasis [8,9]. Clinical studies have shown that serum IGF-1 levels are dysregulated in breast [10], ovarian [11], pancreatic [12], and gastric cancers [13].

IGFBP-4 is a member of the IGFBP protein family. IGFBP-4 regulates the interactions between IGF ligands and cell surface receptors, thereby promoting cell proliferation [14]. IGFBP-4 expression has been shown to be associated with cancer types such as lung cancer [15], epithelial ovarian cancer [16], and glioblastomas [17]. Recent studies have shown that expression levels of IGFBP-4 are significantly higher in gastric cancer tissues [18].

IGFBP-5 is another member of the IGFBP family and has proliferation-inhibiting properties [19,20]. IGFBP-5 exhibits dual behavior across different types of cancers; for instance, while it exerts both anti-tumorigenic and pro-tumorigenic effects on breast and ovarian cancers, its expression is elevated in glioblastoma and colon cancers, suggesting a predominantly pro-tumorigenic behavior in these types of cancers [21].

PAPP-A, an enzyme that facilitates the release of IGF-1 and which binds to IGFBP-2, -4, and -5, is a member of the Pappalysin subfamily of the zinc metalloproteinase family [22]. PAPP-A is considered the primary protease of IGFBP-4 [23]. PAPP-A expression has been shown to be increased in breast [24], lung [25], and ovarian cancers [26]. PAPP-A has been reported to be overexpressed in gastric cancer tissues and associated with poor prognosis. These findings suggest that PAPP-A may be a potential biomarker and therapeutic target involved in tumor progression. Moreover, differential gene expression analyses also found that high *PAPP-A* expression was associated with an increase in IGF-1 and IGFBP-4 in gastric cancer [27,28].

The IGF signaling pathway is complex due to its interactions with multiple ligands. Therefore, understanding the protein–protein interactions of this pathway and the biochemical pathways it influences is significant in cancer studies. Based on our literature review, we found no in vivo studies on PAPP-A in gastric cancer. Although IGFBP-4 and IGFBP-5 have been investigated in bioinformatic and genetic analyses [22,23], to our knowledge, there are no in vivo studies that have assessed serum samples.

Based on this, we aimed to evaluate the IGF signaling pathway as a potential gastric cancer biomarker and to investigate the serum levels of these molecules in different histological types of gastric cancer.

## 2. Results

### 2.1. ELISA Results

**Figure 1:**
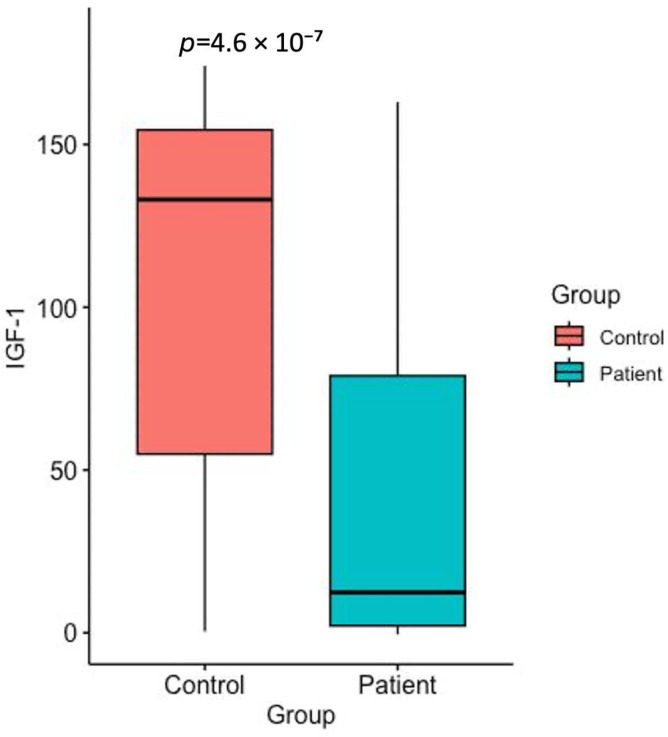
Serum IGF-1 levels in the healthy control group and the gastric cancer patients (*p* < 0.05).

**Figure 2:**
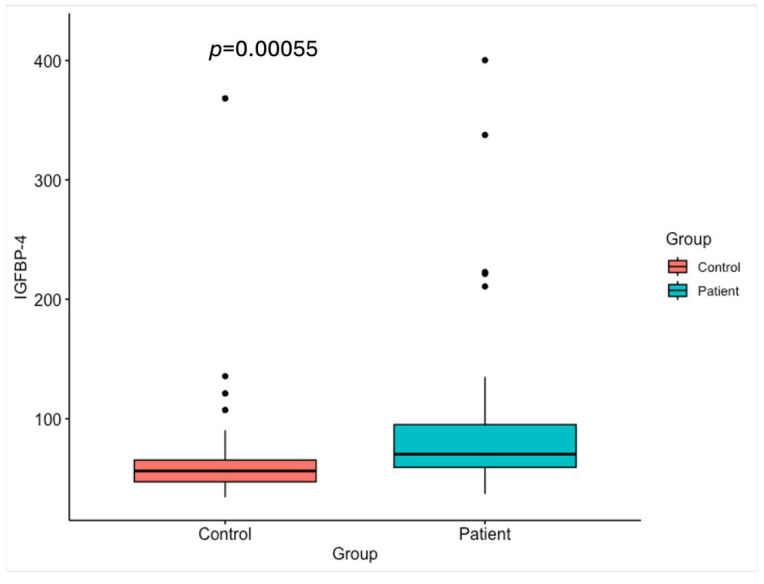
Serum IGFBP-4 levels in the healthy control group and the gastric cancer patients (*p* < 0.05).

**Figure 3:**
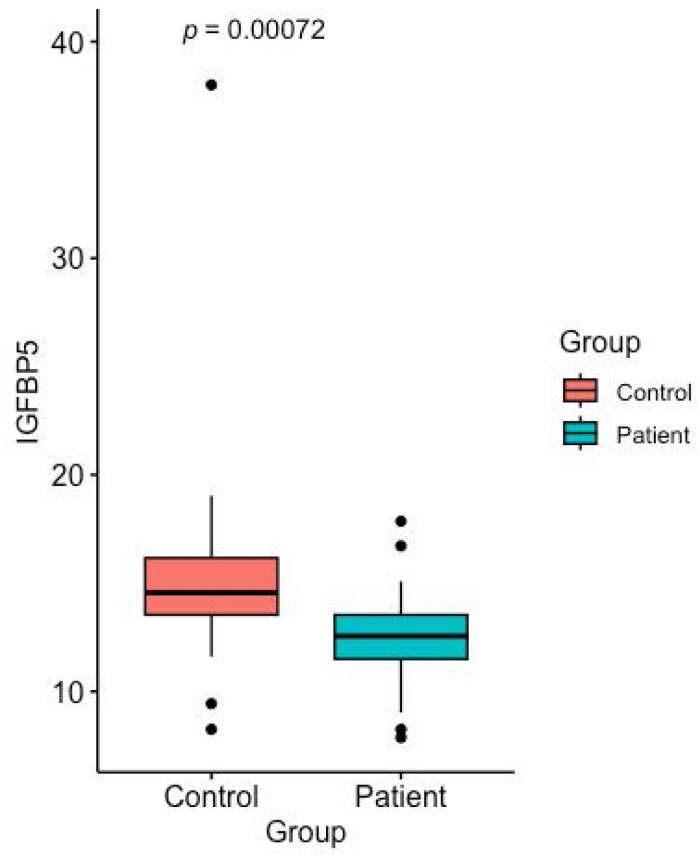
Serum IGFBP-5 levels in the healthy control group and the gastric cancer patients (*p* < 0.05).

**Figure 4:**
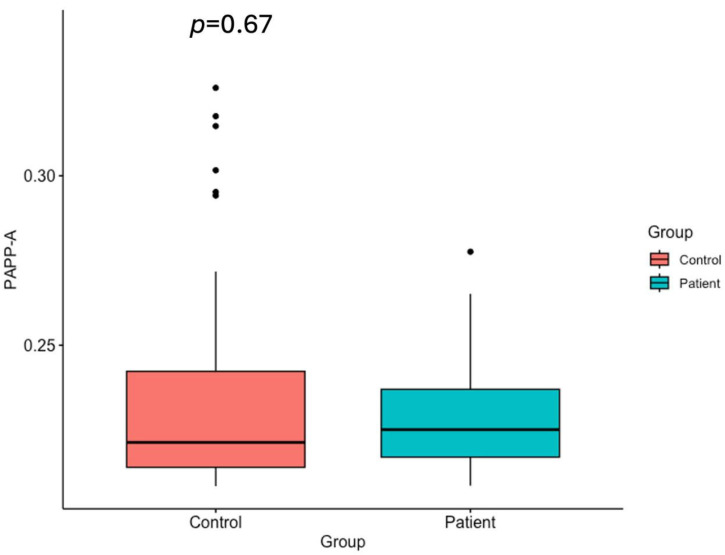
Serum PAPP-A levels in the healthy control group and the gastric cancer patients (*p* = 0.67).

The serum levels of IGF-1, IGFBP-4, IGFBP-5, and PAPP-A in gastric cancer patients and healthy control groups are shown in Table 1 and Figure 1, Figure 2, Figure 3 and Figure 4.

### 2.2. IGF-1 Levels

The serum IGF-1 levels in all gastric cancer patients, regardless of histological differences, were significantly decreased compared to the healthy group (Figure 1) (*p* < 0.05).

### 2.3. IGFBP-4 Levels

The serum levels of total IGFBP-4 protein were significantly higher in gastric cancer patients (97.42 ng/mL) than in healthy individuals (67.88 ng/mL) (Figure 2).

### 2.4. IGFBP-5 Levels

According to the last analysis, the total IGFBP-5 levels showed higher values in the control group than in the patient group. Statistical analysis revealed that this difference was significant (*p* = 0.0035) (Figure 3).

### 2.5. PAPP-A Levels

Although the serum PAPP-A levels in the gastric cancer group (0.228 ng/mL) were lower than those in the group of healthy individuals (0.235 ng/mL), the difference was not statistically significant (Figure 4) (*p* = 0.067).

### 2.6. Receiver-Operating Characteristics (ROC) Analysis

We performed ROC curve analysis of IGF-1, PAPP-A, IGFBP-4, and IGFBP-5 in all 40 patients. In the gastric cancer serum sample, IGF-1 and IGFBP-5 were the most accurate biomarkers in differentiating between gastric cancer patients and healthy individuals. While IGFBP-4 showed a moderately discriminatory effect, PAPP-A had low potential for being a biomarker in gastric cancer detection.

### 2.7. IGF-1

**Figure 5:**
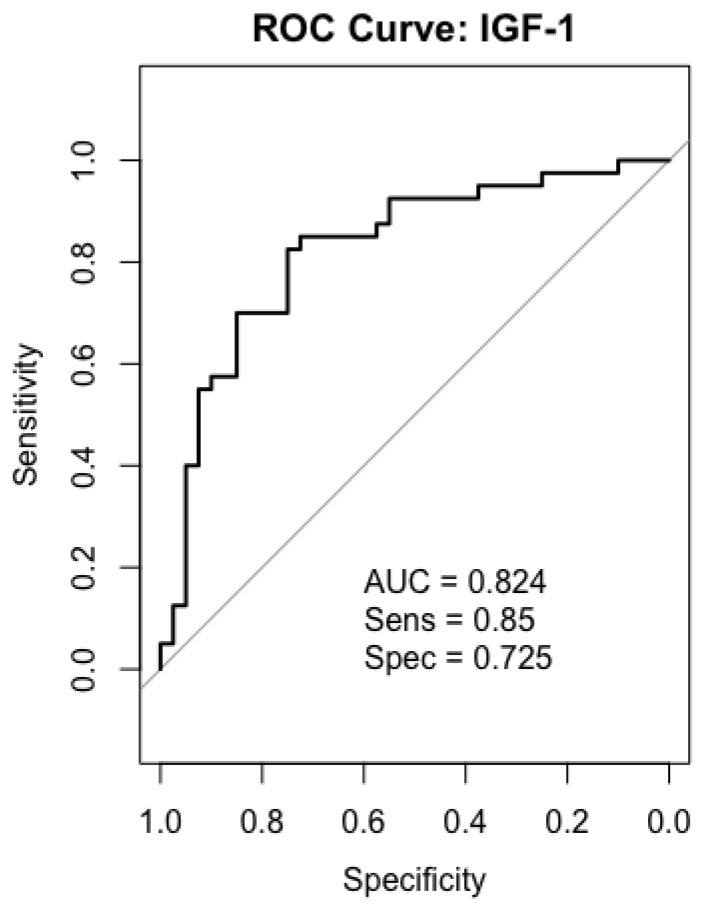
ROC curves analysis for IGF-1 in gastric cancer patients.

As a result of ROC analysis, the AUC value obtained for IGF-1 was 0.824, which shows that IGF-1 has a good diagnostic power in distinguishing between patients and healthy individuals with a sensitivity of 85% and specificity of 72–75% (Figure 5).

### 2.8. IGFBP-4

**Figure 6:**
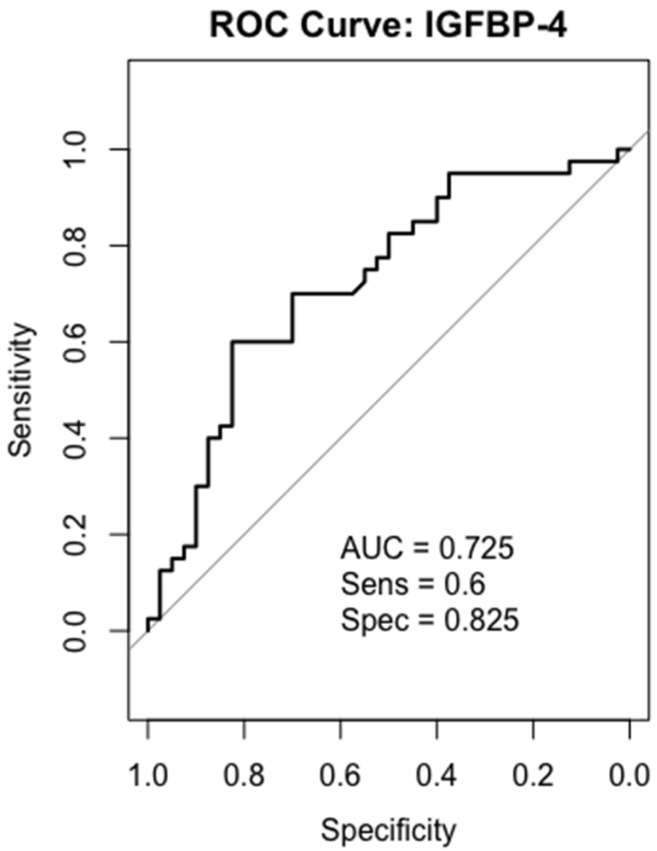
ROC curves analysis for IGFBP-4 in gastric cancer patients.

The calculated AUC value for IGFBP-4 was found to be 0.725, demonstrating moderate discrimination. While its high specificity (82.5%) is particularly advantageous in reducing the false-positive rate, it is thought that some patients may be missed due to its relatively low sensitivity (60%) (Figure 6).

### 2.9. IGFBP-5

**Figure 7:**
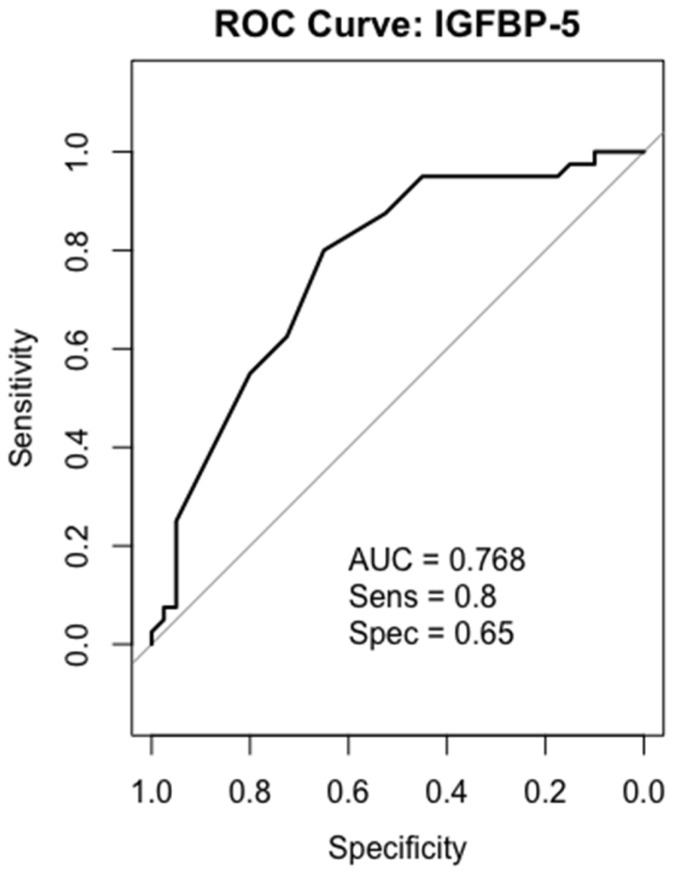
ROC curves analysis for IGFBP-5 in gastric cancer patients.

ROC analysis for IGFBP-5 yielded an AUC of 0.768, indicating good discrimination. While high sensitivity (80%) provides an advantage in accurately identifying diseased individuals, relatively low specificity (65%) can increase the false-positive rate (Figure 7).

### 2.10. PAPP-A

**Figure 8:**
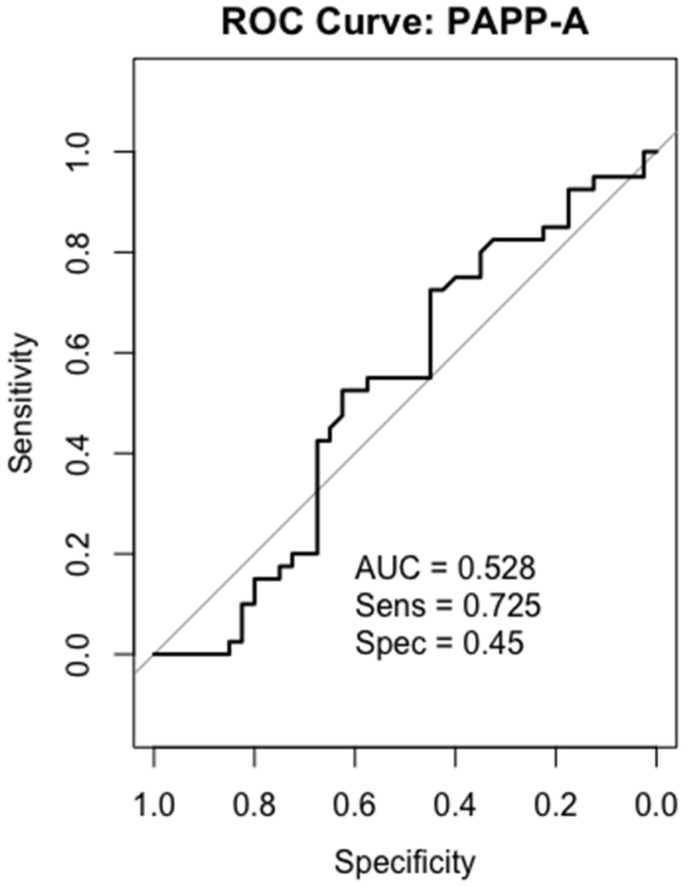
ROC curves analysis for PAPP-A in gastric cancer patients.

The AUC value for PAPP-A was 0.528, which was close to the random guess level. Due to its low specificity (45%) and low AUC, it was concluded that PAPP-A alone does not have diagnostic value as a reliable biomarker (Figure 8).

## 3. Discussion

The IGF signaling pathway has been implicated in cancer biology, yet its role remains controversial, with studies reporting both oncogenic and tumor-suppressive functions depending on the type of cancer. In this study, serum levels of IGF signaling pathway-related proteins known as IGF-1, IGFBP-4, IGFBP-5, and PAPP-A were compared between gastric cancer patients and healthy controls. Our results indicated two key observations: (i) Statistically significant differences were found in the levels of IGF-1, IGFBP-4, and IGFBP-5. (ii) Serum levels of PAPP-A did not show a significant difference between the groups. These results suggest that IGF-1, IGFBP-4, and IGFBP-5 may have potential as diagnostic biomarkers for gastric cancer. Moreover, ROC analysis showed that IGF-1, IGFBP-4, and IGFBP-5 could effectively distinguish gastric cancer from healthy controls with high sensitivity and specificity.

One of the hallmarks of tumor growth is overexpression of growth factors and their receptors. IGF-1 is a well-known growth factor that has a role in cell signaling and progression [8,29]. To date, studies on gastric cancer and IGF-1 have yielded inconsistent results. Serum IGF-1 levels were considerably lower in patients than in controls in a study that included 20 patients with gastric cancer and 20 healthy controls. This finding supports the idea that IGF-1 dysregulation plays a role in the pathophysiology of gastric cancer [30]. In another study of 26 patients with gastric adenocarcinoma (M/F = 15/11, mean age 65 years), serum IGF-1 levels were measured. All gastric cancer patients had higher IGF-1 serum levels compared with healthy participants. There was a significant decrease in IGF-1 levels after surgery (day 14), and this decrease was still present late in the postoperative period (day 50) [31]. In our study, IGF-1 levels were significantly lower in gastric cancer patients compared to the healthy control group. Similarly, in a meta-analysis conducted in 2021, IGF-1 levels were lower in gastric cancer patients than in healthy individuals [32]. The decrease in IGF-1 in serum is the combined result of two distinct mechanisms. In tumor tissue, the IGF-1/IGF-1R interaction may be increased, activating intracellular PI3K/AKT and MAPK signaling pathways, increasing proliferation, and causing local depletion of IGF-1. At the same time, elevated IGFBPs in the circulation (serum) bind IGF-1, thereby reducing its biologically active fraction. In other words, the decrease in serum levels is due to both excess utilization in the tissue and binding in the circulation; both mechanisms lead to the same result (decreased serum levels) but operate in different ways. These mechanisms may explain why IGF-1 expression appears elevated in gastric cancer tissues [13,31] while it is decreased in serum. These findings also support the clinical utility of IGF-1 as a potential biomarker, as reflected by its significant AUC value (0.824) in the ROC analysis.

IGFs bind to their IGFBPs in order to initiate signaling [33]. Among these, IGFBP-4 can bind to IGFs with high affinity and inhibit their function [17]. IGFBP-4 serum levels were found to be higher in breast cancer [34], epithelial ovarian cancer [16], and lung cancer [35]. However, there is no clinical study on serum IGFBP-4 levels in patients with gastric cancer. Yi et al. investigated IGFBPs in 11 gastric cancer cell lines. Overexpression of *IGFBP-4* was observed in all cell lines [36]. Similarly, a bioinformatic study demonstrated increased IGFBP-4 levels in gastric cancer tissues [18]. In a study conducted with 375 cancer samples, it was also found that IGFBP-4 levels were higher in gastric cancer tissues and MKN28, MKN45, HGC27, BGC823, MGC803 and AGS gastric cell lines [28]. In line with previous findings, in our study, IGFBP-4 levels were also increased in gastric cancer serum samples. The results of our study suggest that IGFBP-4 may have a potential role in gastric cancer diagnosis. As previously mentioned, IGF-1 is a potent mitogenic factor that supports cell proliferation, differentiation, and metastatic processes. Therefore, low serum levels may seem contradictory at first glance. However, this situation is thought to be due to increased consumption in tumor tissue [13], binding via IGFBPs and decreasing bioavailability, or systemic metabolic reprogramming. The high IGFBP-4 levels in our study, in particular, suggest that IGF-1 may be present in a more bound form in the circulation, leading to a decrease in its active unbound fraction. This finding indicates that the increase in IGFBP-4 may contribute to reduced IGF-1 bioavailability in serum. Although the ELISA kit used measures total IGF-1, increased circulating IGFBP-4 levels may lead to increased IGF-1 binding and a decrease in its biologically active portion. Moreover, ROC analysis has shown that IGFBP-4 could serve as a moderate biomarker of gastric cancer diagnosis with an AUC of 0.725. Nevertheless, although our findings suggest a possible role for IGFBP-4 in the pathophysiology of gastric cancer, the lack of separate measurement of intact IGFBP-4 levels limits the ability to directly assess the relationship between PAPP-A-mediated proteolysis and IGF bioavailability.

IGFBP-5 also regulates the IGF signaling pathway by binding to IGF-1 [37]. Although studies showing the relationship between serum IGFBP-5 levels and cancer are limited, while IGFBP-5 expression has been reported to increase in pancreas tissues [38], it has also been demonstrated that low expression of IGFBP-5 in breast [39], renal [40] and lung [41] cancer tissues [21]. These results depict the dual effects of IGFBP-5 on different cancer types. In addition to that, IGFBP-5 has been identified in the literature as a binding protein with proliferation-suppressing properties and is known to have anti-tumor effects [42,43]. In a study conducted by Liu et al. IGFBP-5 expression was found to be significantly lower in tumor tissues compared to normal tissues. This was thought to be associated with poor prognosis and may play a role in tumor progression through extracellular matrix-mediated mechanisms [18]. It has also been reported that IGFBP-5 may play a tumor-suppressing role in gastric cancer. PKNOX2 has been shown to activate the p53 pathway by increasing IGFBP-5 expression, suppressing proliferation and increasing apoptosis, as well as inhibiting the epithelial to mesenchymal transition process [44]. In another study using 11 gastric cancer cell lines, IGFBP-5 was expressed in 50% of the cells [36]. Bioinformatics analysis also demonstrated significantly lower IGFBP-5 expression in gastric cancer tissues [18,45]. A study in gastrointestinal stromal tumor models reported that silencing DOG1 significantly increased IGFBP-5 expression, and this increase may be related to suppression of the IGF pathway. This finding indicates the tumor suppressor potential of IGFBP-5 in various gastrointestinal tumors and suggests that it may play a similar role in the context of gastric cancer [46]. Consistent with these observations, our study revealed that total IGFBP-5 serum levels were significantly lower in gastric cancer patients compared to healthy controls. Therefore, the low IGFBP-5 levels in our study might indicate a loss of this inhibitory effect in gastric cancer. ROC analysis further showed that IGFBP-5 can discriminate gastric cancer patients from healthy individuals with an AUC of 0.768, supporting its potential as a clinically related biomarker.

The literature has reported that PAPP-A is increased in breast [27], lung [47], and ovarian cancers [48], whereas it has been found to be decreased in renal cell carcinoma tissues and may act as a tumor suppressor [49]. Current evidence suggests that PAPP-A is overexpressed at the tissue level in gastric cancer and may be associated with adverse clinical parameters [28]. In a study conducted by Liu et al. [18], PAPP-A was found to be associated with overall survival in gastric cancer. However, evidence from serum-based in vivo studies in gastric cancer is quite limited. Thus, investigating circulating PAPP-A levels in gastric cancer would fill a significant gap in the literature. In our study, PAPP-A levels did not show a significant difference between the groups. This contrasts with increases reported in the literature. There may be several reasons for this, as follows: (1) The increase in PAPP-A may occur at the tissue level and therefore may not be reflected in serum. (2) The role of PAPP-A’s proteolytic activity in the local microenvironment may lead to a lack of significant differences in systemic circulation. (3) The limited number of patients may have led to the failure to statistically demonstrate small differences. Moreover, the use of PAPP-A as a serum biomarker alone appears limited, with an AUC of 0.528.

**Figure 9:**
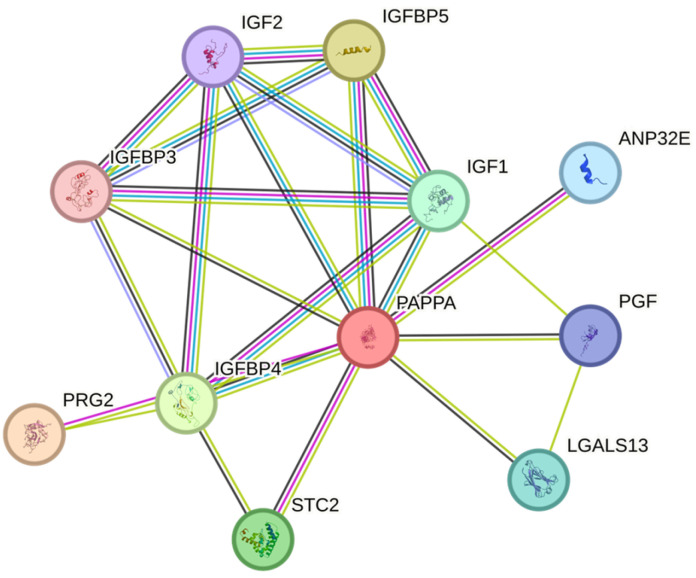
Network map of IGF signaling Pathway Components in STRING Database.

The study was evaluated using the STRING database (Figure 9). IGF-1, IGFBP-4, and IGFBP-5 were found to be involved in a highly connected interaction network. IGFBP-4 and IGFBP-5, in particular, directly interact with IGF ligands and receptors and are linked to bioavailability regulation via PAPP-A. Network analysis revealed that these molecules do not operate in isolation but rather in conjunction with a functional module. This perspective supports the notion that the changes observed in serum levels are not independent of each other but are the result of imbalances within the same biological network. Furthermore, STRING analysis reveals that poorly investigated proteins such as LGALS13 and PGF also appear to peripherally interact with the IGF axis. The role of these molecules in gastric cancer is not yet clear, but they are potential candidates that could shed light on future research.

These changes in the IGF axis are directly linked to the fundamental characteristics of cancer. A decrease in IGF-1 and an increase in IGFBP-4 may be indicative of enhanced proliferation, while a decrease in IGFBP-5 may be indicative of reduced apoptosis. Furthermore, IGF-1R activation triggers cell migration, invasion, and metastasis via the PI3K/AKT/mTOR and MAPK pathways. This is consistent with the hallmarks of “sustaining proliferative signaling,” “resisting cell death,” and “activating invasion and metastasis” [50]. Our study supports this biological framework based on clinical serum levels.

This study has some limitations. The most important restriction of our study was the small number of patients, the heterogeneity of histological subtypes, and the measurement of total protein levels, especially for IGFBP-4 and IGFBP-5, which did not distinguish between intact and fragmented samples. Although we determined the biomarker levels in different stages of gastric cancer, we did not detect significant differences. Subgroup comparisons according to histological subtypes were not performed due to the unequal distribution of cases.

Overall, these results demonstrate that the IGF signaling pathway plays a critical role in the pathogenesis of gastric cancer and that IGF-1, IGFBP-4, and IGFBP-5, in particular, may serve as potential biomarkers for clinical applications. STRING analysis reveals that these molecules are closely interconnected and should be evaluated together. These results highlight the importance of the IGF axis in gastric cancer biology and suggest that it may provide guidance for future diagnosis, prognostic assessment, and even targeted therapies. Prospective studies with larger sample sizes will further demonstrate the utility of these biomarkers in early diagnosis and prognosis.

## 4. Materials and Methods

### 4.1. Study Population

It has been reported that the incidence of gastric cancer is lower in individuals under 35 years of age, regardless of gender, while cardiovascular, diabetes, and kidney diseases are more common in those over 65 years of age, and this may affect IGF-1 and PAPP-A levels [24,25,26,27]. Furthermore, PAPP-A levels are known to physiologically increase during pregnancy [51]. Cancer patient samples were obtained from Istanbul University, Faculty of Medicine, considering the following exclusion criteria:

1. Patients who had undergone chemotherapy or surgery for stomach cancer were excluded, as this was thought to affect the study results. Only individuals with a diagnosis were collected. 

2. Individuals under 45 and over 65, as well as those with diabetes mellitus, cardiovascular disease, chronic renal failure, and pregnancy, were excluded from the study.

The study was conducted at Acibadem Mehmet Ali Aydinlar University and at the Istanbul University, Institute of Oncology and Department of Emergency Surgery, Faculty of Medicine. A total of 80 participants, 40 healthy and 40 gastric cancer patients, were included in this study. The patient population of the study comprised individuals with different histological subtypes, such as adenocarcinoma, intestinal type carcinoma, diffuse type carcinoma, neuroendocrine carcinoma and others, and various stages (I–IV) of gastric cancer. Detailed characteristic information about the participants is shown in Table 2 and Table 3.

The study was approved by the Ethics Committee of Acibadem Mehmet Ali Aydinlar University (ATADEK). Ethical approval was first approved in 2022 (Approval number: 2022-14/02) and renewed with amendments in 2024 (Approval number: 2024-9/336). Every participant in the research provided written informed consent.

### 4.2. Measurement of the IGF Components by Enzyme-Linked Immunoassay (ELISA)

A total of 8 mL of blood samples were collected from participants after an overnight fast. The samples were centrifuged at 4000× *g* for 10 min. Serum samples were aliquoted and stored at −80 °C until analysis.

The total IGFBP-4 levels were measured using a sandwich ELISA assay (AnshLabs, LLC, Webster, TX, USA) kit, while PAPP-A, IGF-1 and total IGFBP-5 levels were measured using the Elabscience (Elabscience Biotechnology Co., Ltd., Houston, TX, USA; and AnshLabs, Webster, TX, USA) kits using the sandwich ELISA method. Absorbance values were measured at 450 nm using a microplate reader (BioTek Instruments, Inc., Winooski, VT, USA).The lower limit of detection (LOD) for IGFBP-4 was 4.735 ng/mL, and the total coefficient of variation (CV) was 3.81% for Control I (122.33 ng/mL) and 3.85% for Control II (363.28 ng/mL). The LOD for PAPP-A was 0.47 ng/mL, and the measuring range was 0.78–50 ng/mL. The intra-assay CV was reported as 4.79–6.90%, and the inter-assay CV was reported as 7.45–8.69%. For IGF-1, the LOD of the assay was 0.94 ng/mL, and the measuring range was 1.56–100 ng/mL. The intra-assay CV was 5.97–6.38%, and the inter-assay CV was indicated as 7.06–8.44%. For IGFBP-5, the LOD of the assay was 0.94 ng/mL, and the measuring range was 1.56–100 ng/mL; intra-assay CV was 4.01–6.18%, and the inter-assay CV was 7.28–8.93%.

### 4.3. Statistical Analysis

Data were analyzed using R Software (version 4.3.2; R Foundation for Statistical Computing, Vienna, Austria). The Shapiro–Wilk test was used to determine data normality. Statistical difference analyses were performed using the nonparametric Mann–Whitney U test. ROC (Receiver Operating Characteristic) curve analysis was performed to evaluate the diagnostic performance of IGF-1, IGFBP-4, IGFBP-5, and PAPP-A in distinguishing between patient and healthy groups. The area under the curve (AUC) was calculated with 95% confidence intervals. Cut-off values were determined. Sensitivity and specificity values were defined according to this threshold value. A *p*-value < 0.05 was considered statistically significant.

## 5. Conclusions

In this study, IGF-1, IGFBP-4, IGFBP-5, and PAPP-A levels were examined in serum samples obtained from gastric cancer patients and healthy individuals. Late diagnosis of gastric cancer is one of the main reasons for increased mortality rates. Therefore, identifying reliable biomarkers is crucial for early diagnosis and disease monitoring. While studies highlight PAPP-A as a potential biomarker in the literature, our results revealed significant differences in IGFBP-4 levels in gastric cancer patients. This finding suggests that IGFBP-4 may play a role in cancer biology through not only IGF-dependent but also IGF-independent mechanisms.

In conclusion, among the IGF family members examined, IGFBP-4 may be a more potent and innovative biomarker candidate for gastric cancer. However, the clinical potential of this molecule requires further validation with larger patient groups, multicenter, and prospective studies. Further studies, particularly those considering different histological subtypes and disease stages, will more clearly reveal the potential contributions of IGFBP-4 in diagnosis, prognosis, and treatment follow-up.

## Figures and Tables

**Table 1:** Serum levels of IGF-1, IGFBP-4, IGFBP-5, and PAPP-A in patients with gastric cancer and in the normal healthy control group. Data are shown as median, interquartile range (IQR) ± standard deviation (SD).

	Control (*n* = 40)	Cancer (*n* = 40)
	Median (IQR) ± SD	Median (IQR) ± SD
IGF-1 (ng/mL)	133.1 ± 56.0 (54.9–154.5)	12.37 ± 50.4 (2.1–78.9)
IGFBP-4 (ng/mL)	56.2 ± 53.1 (47.2–65.4)	70.3 ± 77.0 (59.3–95.0)
IGFBP-5 (ng/mL)	14.56 ± 4.2 (13.54–16.16)	12.56 ± 2.0 (11.50–13.54)
PAPP-A (ng/mL)	0.221 ± 0.03 (0.214–0.242)	0.225 ± 0.016 (0.217–0.237)

**Table 2:** Characteristics of Participants.

	Cancerous	Healthy
*n*	40	40
Age, m (max–min)	58 (45–65)	53 (45–65)
Male/Female	29/11	32/8

**Table 3:** Characteristics of Gastric Cancer Patients.

Variable	*n*	Percentage (%)
Histology		
Adenocarcinoma	21	26.0
Intestinal type	2	19.5
Diffuse type	15	2.7
Neuroendocrine tumor	1	2.4
Other	1	2.4
**Stages**		
Stage IV	14	35
Stage III	14	35
Stage II	11	27.5
Stage I	1	2.5

## Data Availability

The data supporting the findings of this study are available from the corresponding author upon reasonable request. Due to privacy and ethical restrictions, the data are not publicly available.

## Abbreviations

The following abbreviations are used in this manuscript:

GC	Gastric cancer
IGF	Insulin-like growth factor
PAPP-A	Pregnancy-associated plasma protein A
IGFBP	Insulin-like growth factor binding protein
ELISA	Enzyme-linked immunoassays
CV	Coefficient of variation
LOD	Limit of detection
